# Editorial: Community series: police trauma, loss, and resilience, volume II

**DOI:** 10.3389/fpsyg.2024.1348111

**Published:** 2024-05-10

**Authors:** Michael David Schlosser, Konstantinos Papazoglou, Katy Kamkar

**Affiliations:** ^1^University of Illinois at Urbana-Champaign, Champaign, IL, United States; ^2^ProWellness Inc., Woodbridge, ON, Canada; ^3^The POWER Project, San Diego, CA, United States; ^4^University of Toronto, Toronto, ON, Canada

**Keywords:** police officer, officer wellness, resilience, trauma, mental health

Policing is a profession marked by myriad challenges, where officers regularly confront traumatic incidents, grapple with loss, and endeavor to maintain resilience amid adversity. As custodians of public safety, law enforcement personnel face a spectrum of stressors that profoundly impact their mental and emotional wellbeing. The second volume of the Community Series explores these critical issues, offering valuable insights and perspectives from frontline officers, mental health professionals, and researchers. The following are the titles and a brief description of these crucial articles included in *Community series: police trauma, loss, and resilience – Volume II*.

*Cardioautonomic lability assessed by heart rate variability changes in Royal Canadian Mounted Police cadets during the cadet training program*. This study examines variations in cardioautonomic lability during the Royal Canadian Mounted Police (RCMP) Cadet Training Program, revealing potentially important differences in heart rate variability between cadets with and without clinically significant anxiety symptoms (Teckchandani T. A. et al.).

*Daily survey participation and positive changes in mental health symptom scores among Royal Canadian Mounted Police Cadets*. Investigating the impact of daily survey participation on mental health symptom scores among RCMP cadets, this research highlights the positive correlation between daily survey completion and reductions in symptoms related to various mental health disorders (Shields et al.).

*Prophylactic relationship between mental health disorder symptoms and physical activity of Royal Canadian Mounted Police Cadets during the cadet training program*. Examining the relationship between physical activity and mental health disorder symptoms among RCMP cadets, this study underscores the significant role of physical activity in reducing symptoms of anxiety-related disorders during the training program (Teckchandani T. et al.).

*Examining mental health knowledge, stigma, and service use intentions among Royal Canadian Mounted Police cadets*. This study investigates mental health knowledge, stigma, and service use intentions among RCMP cadets, revealing higher levels of mental health knowledge and service use intentions among cadets compared to serving RCMP officers, with implications for reducing stigma and promoting help-seeking behaviors (Andrews et al.).

*Hostage negotiator resilience: a phenomenological study of awe*. Exploring awe as a resilience practice for law enforcement crisis and hostage negotiators, this research demonstrates how reflecting on awe experiences positively impacts negotiators in both their professional and personal lives, suggesting potential interventions to enhance resilience (Thompson and Jensen).

*Attitudes toward organizational change and their association with exhaustion in a sample of Italian police workers*. This study examines attitudes toward organizational change and their association with exhaustion among Italian police workers, emphasizing the importance of addressing workplace aggression to enhance job satisfaction and wellbeing among officers (Colombo et al.).

*Mental health and social support among Royal Canadian Mounted Police Cadets*. Investigating the relationship between perceived social support and symptoms related to mental disorders among RCMP cadets, this research highlights the protective role of social support against anxiety-related disorders among cadets, with implications for promoting psychological wellbeing (Nisbet et al.).

*Putative risk and resiliency factors among Royal Canadian Mounted Police Cadets*. This study compares putative risk and resiliency factors among RCMP cadets to young adult populations, suggesting that cadets may possess psychological strength, implying that the nature of police work rather than inherent individual differences might contribute to the higher prevalence of mental health disorders among active-duty RCMP officers (Khoury et al.).

*Workplace aggression, wellbeing, and job satisfaction: the specificity in border police organizations*. This study explores workplace aggression and its impact on wellbeing and job satisfaction among Romanian Border Police officers. Findings suggest that workplace aggression negatively predicts job satisfaction and wellbeing, underscoring the need for interventions to reduce such behaviors (Antón et al.).

Through empirical research, case studies, and firsthand accounts, these articles collectively illuminate the multifaceted nature of police trauma, loss, and resilience, offering valuable insights to inform interventions, support systems, and cultural shifts within law enforcement communities. As we navigate these critical issues, let us strive to create a safer, healthier, and more resilient future for all those who serve.

## Author contributions

MS: Writing – original draft, Writing – review & editing. KP: Writing – original draft, Writing – review & editing. KK: Writing – original draft, Writing – review & editing.

